# A hybrid invisibility cloak based on integration of transparent metasurfaces and zero-index materials

**DOI:** 10.1038/s41377-018-0052-7

**Published:** 2018-08-15

**Authors:** Hongchen Chu, Qi Li, Bingbing Liu, Jie Luo, Shulin Sun, Zhi Hong Hang, Lei Zhou, Yun Lai

**Affiliations:** 1 School of Physical Science and Technology and Collaborative Innovation Center of Suzhou Nano Science and Technology, Soochow University, 215006 Suzhou, China; 2 State Key Laboratory of Surface Physics and Key Laboratory of Micro and Nano Photonic Structures (Ministry of Education), Fudan University, 200433 Shanghai, China; 3 Collaborative Innovation Center of Advanced Microstructures, 210093 Nanjing, China; 4 Shanghai Engineering Research Center of Ultra-Precision Optical Manufacturing, Green Photonics and Department of Optical Science and Engineering, Fudan University, 200433 Shanghai, China; 5 State Key Laboratory of Applied Optics, Changchun Institute of Optics, Fine Mechanics and Physics, Chinese Academy of Sciences, 130033 Changchun, China; 6 National Laboratory of Solid State Microstructures, School of Physics, Nanjing University, 210093 Nanjing, China

## Abstract

The invisibility cloak, a long-standing fantastic dream for humans, has become more tangible with the development of metamaterials. Recently, metasurface-based invisibility cloaks have been proposed and realized with significantly reduced thickness and complexity of the cloaking shell. However, the previous scheme is based on reflection-type metasurfaces and is thus limited to reflection geometry. In this work, by integrating the wavefront tailoring functionality of transparent metasurfaces and the wave tunneling functionality of zero-index materials, we have realized a unique type of hybrid invisibility cloak that functions in transmission geometry. The principle is general and applicable to arbitrary shapes. For experimental demonstration, we constructed a rhombic double-layer cloaking shell composed of a highly transparent metasurface and a double-zero medium consisting of dielectric photonic crystals with Dirac cone dispersions. The cloaking effect is verified by both full-wave simulations and microwave experimental results. The principle also reveals exciting possibilities for realizing skin-thick ultrathin cloaking shells in transmission geometry, which can eliminate the need for spatially varying extreme parameters. Our work paves a path for novel optical and electromagnetic devices based on the integration of metasurfaces and metamaterials.

## Optics: Another step closer to an invisibility cloak

Chinese scientists have developed an innovative technique for making an invisibility cloak that could pave the way for new optical and electromagnetic devices. Although invisibility cloaks by bending light around an object have been around for some time, they are bulky and complex devices with spatially gradient parameters, limiting their uses. Now, Yun Lai and his colleagues from Nanjing University and Soochow University, in collaboration with researchers from Fudan University, have developed a new technique that combines the properties of metamaterials and metasurfaces to produce an invisibility cloak that is significantly thinner and less complex than currently available. The hybrid device integrates the wavefront tailoring functionality of transparent metasurfaces with the wave tunneling functionality of materials with near-zero-refractive index, and enables a new approach of cloaking by creating the illusion of free space.

## Introduction

The intriguing concept of invisibility cloaks has aroused great enthusiasm^1–17^ in the past decade. However, invisibility cloaks designed by transformation optics^1,2^ are normally bulky devices with complex spatial distributions of material parameters. Recently, metasurfaces, which are ultrathin metamaterials composed of tailored planar microstructures, have demonstrated extraordinary abilities to control the phase, amplitude, and polarization of electromagnetic waves^18–38^. With the use of metasurfaces, the thickness and complexity of invisibility cloaks have been significantly reduced^34–38^. By covering a corrugated surface with a layer of carefully designed metasurface of skin-like thickness, the wavefront of the reflected waves can be properly modified so as to imitate plane waves reflected from a flat surface^34–37^, creating the illusion^8,17^ of a plane mirror^5^. However, this method only works in reflection geometry. For an invisibility cloak that functions in transmission geometry, in which the illusion of free space is created instead of that of a plane mirror, the requirements are completely different. First, the cloak itself should be non-reflecting. Second, the wave energy should be either directed around a hidden space or compensated by using lossy and gain media^38^. These requirements are beyond the capabilities of reflection-type metasurfaces.

Zero-index materials (ZIMs)^39–55^, that is, metamaterials with a near-zero-refractive index, offer extraordinary wave tunneling functionality to direct the electromagnetic wave energy around a hidden space. Such ZIM-assisted wave tunneling phenomena have been demonstrated in channels and bends using various ZIMs, such as metallic metamaterials^42^, waveguides at cut-off frequencies^43^, double-zero media^39,44–54^ (with simultaneous zero permittivity and zero permeability) composed of dielectric photonic crystals with Dirac cone dispersions^46–53^, and doped epsilon-near-zero materials^54^. Nevertheless, in free space, oblique incident waves are reflected due to the zero-refractive index of ZIMs, which disables the wave tunneling functionality. Therefore, an efficient coupling method is required to exploit the full functionalities of ZIMs in free space.

In this work, for the first time, we propose a general principle to integrate transparent metasurfaces and ZIMs into a unique hybrid invisibility cloak that works in transmission geometry. The key functionalities of wavefront tailoring with metasurfaces and wave energy tunneling with ZIMs are combined together to achieve invisibility through an illusion of “free space.” Numerical simulations not only prove the validity of the cloaking effect for almost any shape of cloak but also demonstrate possibilities in realizing skin-thick cloaking shells in transmission geometry, avoiding the need for spatially varying extreme parameters. By constructing a rhombic double-layer cloaking shell that is composed of a highly transparent metasurface and a double-zero medium consisting of dielectric photonic crystals with Dirac cone dispersions, we have experimentally verified the cloaking effect. Our work not only demonstrates a new principle of cloaking, that is, hybrid invisibility cloaks, but also unveils a novel strategy of integrating metamaterials and metasurfaces together for more sophisticated and advanced functionalities that are beyond each of them alone.

## Materials and methods

### Design principle of the hybrid invisibility cloak

**Fig. 1:**
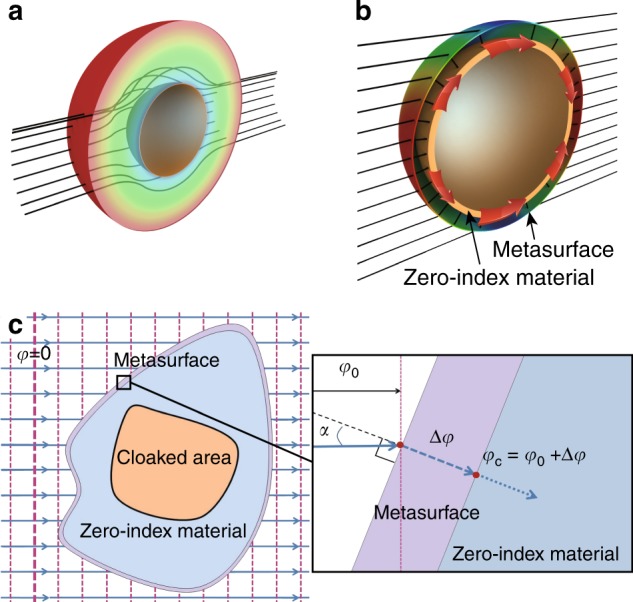
Schematic graphs of the principle of hybrid invisibility cloaks in transmission geometry. **a** Schematic graph of an invisibility cloak based on transformation optics. **b** Schematic graph of a hybrid invisibility cloak based on the integration of metasurfaces and ZIM. The black lines indicate the ray paths of light. A gap between the metasurface and ZIM is enlarged to show the bending of the ray paths. The red arrows indicate the flow of electromagnetic flux inside the ZIM. **c** Sectional view of the cloaking shell of a hybrid invisibility cloak composed of a metasurface covering a layer of ZIM. A zoomed view of the cloaking shell is shown on the right. Here, *α* denotes the local incident angle. *φ*_0_ and *φ*_c_ denote the phases of the incident waves on the metasurface and ZIM, respectively. Δ*φ* denotes the local phase change induced by the metasurface

In the following, we demonstrate the principle of integrating transparent metasurfaces and ZIMs into a single device, hereby denoted as a hybrid invisibility cloak, that can cloak objects in transmission geometry. This fascinating cloaking functionality is realized by combining the wavefront tailoring functionality of metasurfaces and the wave tunneling functionality of ZIMs. Unlike previous invisibility cloaks based on transformation optics^1–17^, which are composed of volumetric gradient structures (Fig. 1a), the cloaking shell of a hybrid invisibility cloak has a simple two-layer design (Fig. 1b). The outer layer is a highly transparent metasurface, and the inner layer is a homogeneous layer of ZIM. The paths of the light rays in Fig. 1b (shown as lines) show the cloaking mechanism. On the entry side, the wavefront of the incident light rays is manipulated by the metasurfaces to be normally incident on the surface of the ZIM, for which the normal incidence guarantees good coupling with the ZIM layer. The ZIM layer guides the wave energy around a hidden space to the exit side, where the light rays are bent back by the metasurface into their original propagating direction. In this way, the wavefront and phase of the incident plane waves can be completely recovered, producing the invisibility of the cloaking shell as well as any objects inside the shell. The arrows inside the ZIM layer indicate the tunneling of the electromagnetic flux around the hidden space. Figure 1c shows the design principle of the hybrid invisibility cloak, with a zoomed view of the cloaking shell shown on the right-hand side. The incident wave is a plane wave propagating to the right. At a local point on the cloaking shell surface, the local incident angle is *α*, and the local phase of the incident wave is *φ*_0_. Without the metasurface, the incident waves are completely reflected when the incident angle *α*≠0 due to the zero-refractive index of the ZIM. However, by covering the ZIM with a carefully designed metasurface, an additional phase Δ*φ* can be imposed on the transmitted wave through each point of the metasurface, changing the phase of the incident wave on the ZIM to *φ*_c_ = *φ*_0_ + Δ*φ*. By designing the metasurface such that the phase *φ*_c_ = *φ*_0_ + Δ*φ* = *c* is a constant on the entire surface of the ZIM, the normal incidence condition of the ZIM is satisfied, and the waves are efficiently coupled into the ZIM. The energy tunneling functionality of the ZIM is enabled, and the wave energy is tunneled to the exit side. On the exit side, the radiated waves from the ZIM are also transformed into the form of a propagating plane wave by the metasurface, which produces the cloaking effect.

## Results and discussion

### Numerical demonstration using the effective medium model

**Fig. 2:**
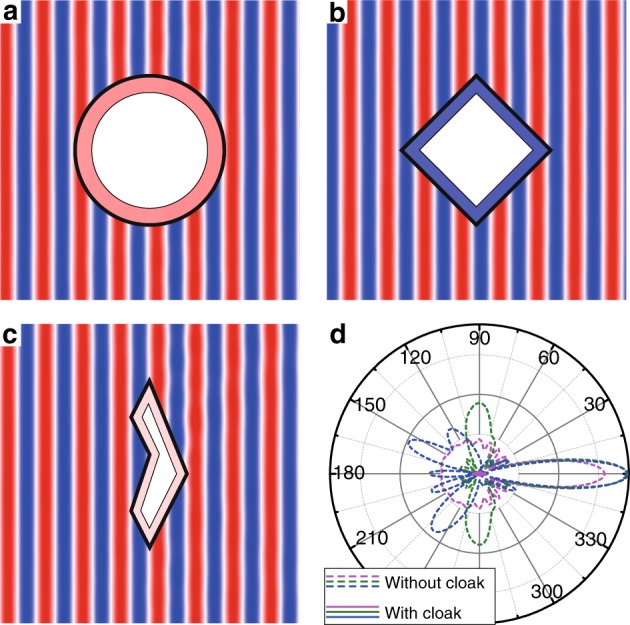
Numerical demonstrations via the effective medium model. **a**–**c** The electric field distributions for the cases of a circular hybrid cloaking shell, a rhombic hybrid cloaking shell, and an irregular hybrid cloaking shell with a complex polygon shape. The illumination source is a TE-polarized plane wave from the left. **d** The far-field radiation patterns of the scattered waves for the cases in **a** (purple), **b** (green), and **c** (blue) with (solid lines) and without (dashed lines) the cloaking shells

We have performed both simulations and experiments to verify this new principle of cloaking based on the integration of metasurfaces and ZIMs. Without loss of generality, we consider a two-dimensional demonstration with transverse electric (TE) polarization. The fundamental idea is proven through finite element simulations using the COMSOL Multiphysics software. The metasurface is represented by an ultrathin layer of an anisotropic effective medium, which exhibits perfect transmission as well as a full range of phase-tuning abilities (see Supplementary Information). For the ZIM, a double-zero effective medium of *ε* = *μ* = 0.001 is applied. The first example is a circular cloak with a hidden region bound by a circular perfect magnetic conductor (PMC) within the double-layer cloaking shell. The simulated electric field distributions under a plane wave incident from the left is calculated with COMSOL Multiphysics and shown in Fig. 2a. It can be observed that there is almost no scattering of waves, indicating a perfect cloaking effect (see movie in Supplementary Information). The metasurface plays the crucial role of matching the incident and radiated plane waves to the ZIM layer in this cloaking phenomenon (see more analysis in Supplementary Information).

Owing to the generality of this principle, the shape of the cloaking device can be almost arbitrary, although its smallest feature size is limited by the unit structure of the metasurface and ZIM. In the second and third examples, a rhombic cloak and an irregular cloak with a complex polygon shape are designed using the same effective medium models for metasurfaces and ZIMs. The corresponding simulated electric field distributions under a plane wave incident from the left are displayed in Fig. 2b, c. It can be observed that good cloaking effects are also achieved even for hybrid invisibility cloaks with such complex shapes. For cloaking in transmission geometry, in addition to near-field distributions, the cloaking effect can also be quantified with far-field radiation patterns, as shown in Fig. 2d for the three cases. The far-field radiation is significantly suppressed by the cloaking shell in all directions for all three cases. The total scattering cross sections are reduced to less than 1% of that in the three cases without the cloaking shells. The detailed parameters of the above three hybrid invisibility cloaks are presented in the Supplementary Information.

### Design and experimental measurement of a rhombic cloaking shell

**Fig. 3:**
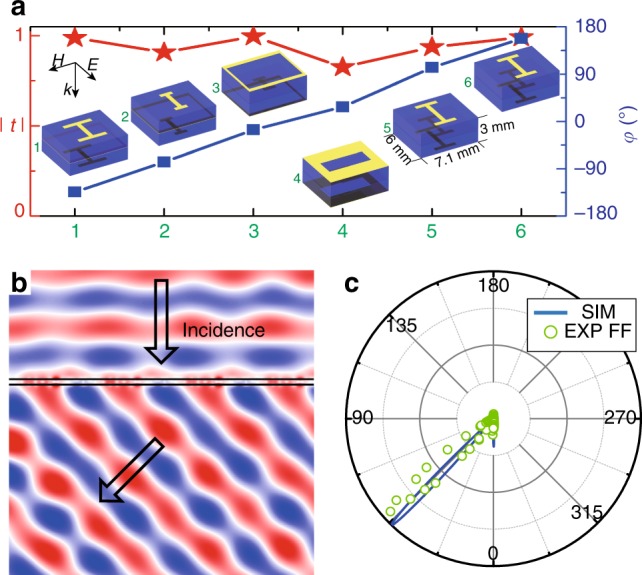
Design and fabrication of the metasurface for a rhombic cloaking shell. **a** Simulated transmission amplitude and phase change of the transmitted waves for the six units of the metasurface. The phase change varies linearly and covers a full range of [0, 2*π*). (Insets) 3D illustrations of six ABA structure units of the metasurface. The yellow parts indicate metallic patterns, while the blue regions indicate two dielectric spacers with a thickness of 1.5 mm. **b** The simulated electric field distribution for the metasurface under the illumination of an incident plane wave with TE polarization. **c** Measured (green dots) and simulated (blue lines) power radiation patterns of the transmitted waves bent by the metasurface at 10.2 GHz

In this section, we further prove the principle of our hybrid invisibility cloak using microwave experiments. We design a rhombic cloak in which the transparent metasurface possesses a linearly varying phase shift along the surface. The metasurface is designed with sandwiched ABA structures, which exhibit good phase-tuning abilities in high transparency conditions^21^. Six ABA metasurface units with a linear phase shift covering the entire range of [0, 2*π*) and high transmittance are configured near 10 GHz. The ABA structures as well as the calculated phase shift and transmittance through each unit are shown in Fig. 3a (with detailed geometric parameters shown in Supplementary Information). By contiguously arranging the six units, we obtain the final metasurface structure of the rhombic cloak. The desired high transparency and beam bending effect of the metasurface are measured to adequately perform from 10 GHz to 10.3 GHz. As shown in Fig. 3b, a plane wave normally incident on the metasurface is bent to the refraction angle of 45° with very little reflection at the frequency of 10.2 GHz, in which the transmittance is measured at 87%. Figure 3c shows the measured (green dots) and simulated (blue lines) power radiation patterns of the transmitted waves. With time reversal, a plane wave incident at 45° is refracted back to the normal direction of the metasurface. These beam bending effects exactly match the requirements of the metasurface of the rhombic cloak. As for the ZIM, we design a photonic crystal that has Dirac cone dispersion at the Brillouin zone center (*Γ* point) in the photonic band diagram. This unique type of photonic crystal effectively functions as a double-zero medium at the frequencies near the Dirac point, which has been previously experimentally verified in both microwave and optical frequencies^46–51^. Here, the designed photonic crystal consists of a square array (with lattice constant of 17.46 mm) of alumina cylinders (with relative permittivity *ε*_r_ = 8.1 and radius 3.95 mm). The frequency of the Dirac point, that is, the frequency at which the photonic crystal behaves as a ZIM, is tuned to be 10.2 GHz (see Supplementary Information), which is compatible with the working frequencies of the designed ABA metasurface.

**Fig. 4:**
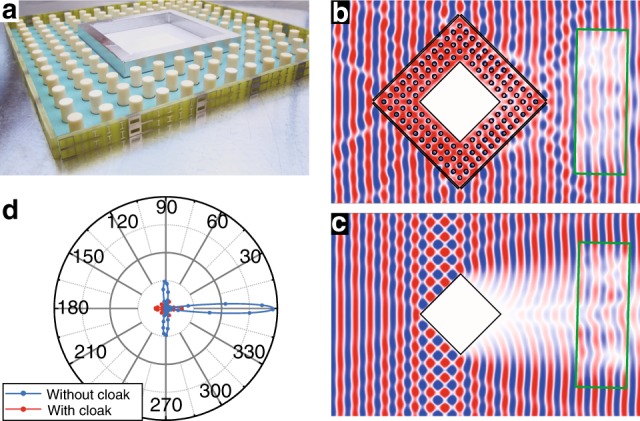
Experimental demonstration of a rhombic cloaking shell. **a** Picture of the fabricated rhombic cloaking shell, which consists of the metasurface shown in Fig. 3 and a layer of ZIM composed of photonic crystals. The central part is a metallic rhombus as the hidden region with a *λ*/4 distance (10.2 GHz) of separation from the photonic crystal. **b** The electric field distribution for the case with the cloaking shell. The part inside the region marked by the green rectangle is experimentally obtained via a microwave field mapper. The other part is obtained by the full-wave simulation. **c** The electric field distribution for the case without the cloaking shell. The parts inside and outside the region marked by the green rectangle are obtained experimentally and numerically, respectively. The sources in (**b**, **c**) are both TE-polarized plane waves incident from the left. **d** Simulated power radiation patterns for a rhombic PEC scatterer with and without cloaks

By combining the designed metasurface and ZIM, a rhombic cloaking shell with a side length of 234.3 mm is realized, as shown in Fig. 4a. The to-be-cloaked region is bound by a rhombic metallic structure with a side length of 108 mm. A distance of *λ*/4 is set between the metallic structure and the photonic crystal ZIM, where *λ* is the wavelength at 10.2 GHz in free space. With this separation, the metallic structure can resemble a PMC. In the microwave experiment, the cloaking device is placed inside a parallel plate waveguide (top plate not shown in Fig. 4a) that allows TE-polarized propagating waves. A horn antenna placed far from the waveguide is used to generate a TE-polarized plane wave inside the waveguide (shown in Fig. S1 in Supplementary Information). Due to the size limitation of the translational stage, the electric field distribution within a rectangular region of 280 × 100 mm^2^ behind the cloaking shell is measured. For reference, full-wave simulations are performed by using the finite-difference-time-domain simulation software CST. Figure 4b shows a combination of electric field distributions obtained by the full-wave simulation and microwave experimental measurement. The experimental results are plotted inside the regions highlighted by a green rectangular frame. The simulation results are plotted outside the green rectangular frame. Good agreement is observed between the simulation and experimental results. Both results clearly demonstrate a good cloaking effect by the rhombic cloaking shell. For comparison, we show the field distribution obtained under the same incident wave for the case without the cloaking shell in Fig. 4c. Again, the simulation and experimental results correspond well. However, in this case, a distorted wavefront is observed on the right-hand side of the rhombic metallic structure (object), which is induced by the scattering effect of the metallic structure. To quantitatively study the cloaking performance of the hybrid cloak, we further calculate the power radiation patterns for the rhombic metallic structure with and without the hybrid cloak, as shown in Fig. 4d. The scattering patterns of the rhombic metallic structure, which are originally largely distributed in the directions of 0°, 90°, and 270° (i.e., the forward, upward, and downward directions) due to the rhombic shape, are significantly suppressed by the hybrid cloak, although the size of the hybrid cloak is almost twice that of the rhombic metallic structure. The scattering of the cloak comes mainly from the imperfection of the metasurface and photonic crystal in both the design and fabrication processes and the corner effect of the rhombic hybrid cloak (~0.15*λ* in this study). Optimization through a more meticulous design or utilization of the concept of Huygens’ metasurface may further reduce the total scattering of the cloak.

### Discussion on the advantages and limits of hybrid invisibility cloaks

**Fig. 5:**
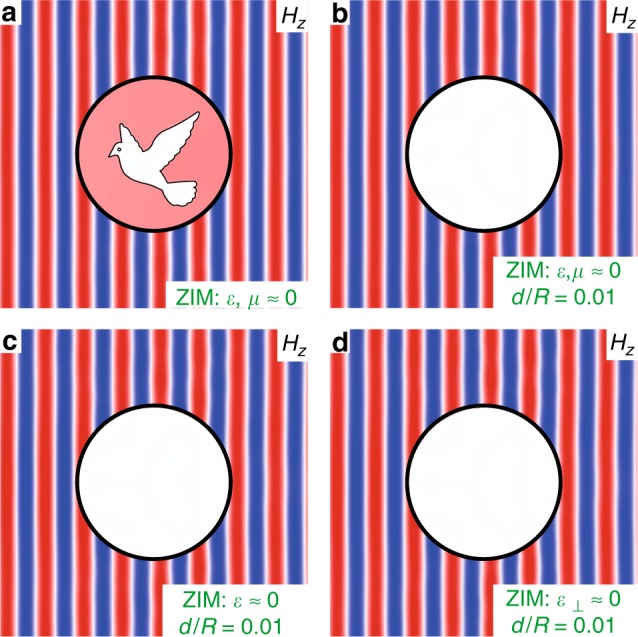
Effective medium simulations of ultrathin hybrid invisibility cloaks and a wide variety of ZIMs. **a**–**d** Snapshots of the simulated magnetic fields for circular hybrid invisibility cloaks with various geometries and types of ZIMs under an incident TM plane wave from the left. **a** A ZIM layer composed of a double-zero medium with *ε* = *μ* = 0.001 embedded with a dove-shaped PEC scatterer. **b** A ZIM layer composed of a double-zero medium with *ε* = *μ* = 0.0001 and thickness *d*_ZIM_ = 0.005*R*. **c** A ZIM layer composed of a single-zero medium with *ε* = 0.0001 and thickness *d*_ZIM_ = 0.005*R*. **d** A ZIM layer composed of an anisotropic zero-index medium with *ε*_⊥_ = 0.0001 and thickness *d*_ZIM_ = 0.005*R*. The parameters and thicknesses (*d*_MS_ = 0.005*R*) of the metasurfaces in (**a**–**d**) are exactly the same

The principle of hybrid invisibility cloaks is numerically and experimentally proved in the previous sections. In this section, we discuss the advantages and limits of this principle. In the following demonstrations, TM (transverse magnetic) polarization is applied instead of TE polarization. First, we note that for a ZIM layer composed of a double-zero medium, there is almost no limit on its shape except that the smallest feature size is determined by the unit structure. For instance, as shown in Fig. 5a, we modify the shape of the double-zero ZIM in the circular cloaking shell (in Fig. 2a), such that the hidden object turns into a dove-shaped scatterer. Near-perfect cloaking functionality is still achieved. The metasurfaces applied here can be conveniently obtained by exchanging the *ε* and *μ* parameters of the metasurfaces in Fig. 2. Interestingly, by significantly reducing the thickness of the ZIM layer, it is, in principle, possible to realize an “ultrathin cloak” in transmission geometry. By “ultrathin cloak”, we imply that *d*/*R*→0, where *d* is the thickness of the cloak and *R* is the radius of the cloaked area. When *d*/*R*→0, traditional transformation optics cloaks require divergent parameters in the cloaking shell (see the Supplementary Information), which make the realization of ultrathin cloaks quite difficult, especially in the optical frequency regime. In contrast, for the hybrid invisibility cloaks designed in this study, the parameters of the cloaking shells remain finite (for both the metasurfaces and ZIMs) even when *d*/*R*→0. This advantage of hybrid invisibility cloaks renders the realization of ultrathin cloaks possible. In Fig. 5b–d, we reduce the thickness of the ZIM layers to *d*_ZIM_ = 0.005*R*, and the cloaking effect is still well maintained. The total thickness of the cloaking shell is *d* = *d*_MS_ + *d*_ZIM_ = 0.01*R*. More interestingly, if the thickness of the ZIM layer is reduced to the subwavelength scale, there are more possibilities for the ZIM^39–42^, including double-zero media (Fig. 5b), single-zero media with *ε*→0 (Fig. 5c), and anisotropic zero-index media with *ε*_⊥_→0 (Fig. 5d). Here, *ε* and *ε*_⊥_ represent the permittivity in the isotropic case and the component of permittivity in the radial direction of the cloak in the anisotropic case, respectively. Figure 5b–d demonstrate almost perfect cloaking effects based on effective medium simulations. Although such effective medium based ZIMs with subwavelength thickness cannot be realized by using the dielectric photonic crystals that we adopted in the proof-of-principle experiment, they may be designed and experimentally realized by metallic metamaterials^42^ and waveguides at cut-off frequencies^43^, which have been experimentally demonstrated to exhibit an excellent wave tunneling functionality. However, further structural optimization is required to maintain good coupling between the metasurface and the ZIM.

In the design of hybrid cloaks, the metasurface plays the crucial role of bridging the propagating waves in free space and the tunneling waves in ZIMs. Without such metasurface, the wave tunneling-based cloaking effect of ZIMs is limited in the waveguide configuration, as has been previously experimentally demonstrated^43^. Moreover, there are stringent requirements in the shape and thickness of ZIMs to match the shape of the wavefront of the incident waves and the phase accumulation in free space, respectively. Interestingly, all the above-mentioned restrictions can be removed via the introduction of metasurfaces, which enables cloaks to have arbitrary shapes, as shown in Fig. 2.

Although the hybrid invisibility cloak designed in this study can have arbitrary shapes and possess an ultrathin thickness, it still has limitations set by the metasurfaces, ZIMs, and general physical laws that limit the full realization of cloaking. Therefore, we provide the possible solutions to solve these limitations. For instance, all types of invisibility cloaks are dependent on the morphology and shape of the cloak, including the hybrid invisibility cloaks designed in this study. Nevertheless, we note that an advantage of the hybrid invisibility cloak is that the parameters of the ZIM layer are almost independent of the morphology or shape of the cloak, which greatly reduces the complexity of the cloak design.

Another limitation is the incident angle. Similar to previously reported metasurface cloaks in reflection geometry^34–38^, the metasurface of the hybrid invisibility cloak is designed for a fixed incident angle, and therefore, the cloaking effect is limited to a certain angle with small tolerance. Interestingly, such a limit in angle may be relieved by employing the recently developed coding/programmable metasurfaces^30,32^ or nonlocal metasurfaces^33^. Coding/programmable metasurfaces in principle can change the metasurface parameters based on the direction of incident waves. Nonlocal metasurfaces inherently exhibit different material parameters for different incident angles. In the Supplementary Information, we demonstrate that by applying the advanced techniques of coding/programmable or nonlocal metasurfaces, wide-angle, and even omnidirectional cloaking effects, in theory, may be realized.

As for the limitation on bandwidth, when phase continuity is required (as for transformation optics cloaks and hybrid invisibility cloaks), the cloaking effect is limited to a narrow frequency regime due to the speed limit of light^16^. To overcome this limitation, one feasible solution is to ignore the requirement of phase continuity and design the cloak in the ray-optics limit, for example, ray-optics cloaking^16^. Such a principle can also be applied to hybrid cloaks. The hybrid invisibility cloaks demonstrate, for the first time, that the functionality of cloaking can be divided into two parts: the import and reconstruction of the light field, and the guiding of light. Here, the metasurface is for the import and reconstruction of the light field, while the ZIM is for the guiding of light. But when phase continuity is no longer required, both the metasurfaces and ZIMs can be replaced by other optical devices with larger bandwidths to enhance the total bandwidth of the cloaking device.

Although in this paper we mainly discuss the scheme of hybrid cloaks in two dimensions for single polarization (TE polarization for Figs. 2 and 4, TM polarization for Fig. 5), the principle can be extended to polarization-independent and three-dimensional (3D) cases. In these extensions, one basic requirement is the design of polarization-independent ZIMs, which has been realized by photonic crystals with dual-polarization Dirac cones^52,53^, or the design of 3D ZIMs, which can be realized by combining the Dirac cones in the in-plane and out-of-plane directions in a single photonic crystal. We note that such dielectric ZIMs can be attained at optical frequencies^47–51^. The other requirement is the design of a metasurface for both polarizations, which has been experimentally realized by adopting symmetrical units in the reflection-type metasurface cloak^36,37^ or other metasurface designs. In the Supplementary Information, we discuss the requirements of metasurfaces and ZIMs in hybrid cloaks for both TE and TM polarizations and demonstrate the cloaking effect for both polarizations by using effective medium-model simulations.

## Conclusions

In conclusion, we have numerically and experimentally demonstrated a unique type of hybrid invisibility cloak based on the integration of metasurfaces and ZIMs as a new principle of cloaking. Unlike previous reflection-type metasurface invisibility cloaks^34–37^ that create the illusion of a “plane mirror,” this hybrid invisibility cloak creates new possibilities for the realization of various-shaped and ultrathin cloaking shells in transmission geometry, which leads to the ideal illusion of “free space.” Hybrid invisibility cloaks, a union between metasurfaces and metamaterials, unveil a novel strategy for realizing advanced optical and electromagnetic devices beyond the capabilities of metasurfaces or metamaterials alone.

## Electronic supplementary material

Supplementary information

Supplementary Movie S1
