# Supplementary material for: A hybrid invisibility cloak based on integration of transparent metasurfaces and zero-index materials
Source: Light Sci Appl. 2018 Aug 15;7:50. doi: 10.1038/s41377-018-0052-7 (PMC6107001; doi:10.1038/s41377-018-0052-7)
Supplement: Supplementary file 1 — Supplementary information [file 41377_2018_52_MOESM1_ESM.doc]

Supplementary information for

**Title:** A hybrid invisibility cloak based on integration of transparent metasurfaces and zero-index materials

**Running Head:** A hybrid design for invisibility cloaks

**Authors**

Hongchen Chu^1^,†, Qi Li^2,3^,†, Bingbing Liu^1^,†, Jie Luo^1^, Shulin Sun^4,5^,*, Zhi Hong Hang^1^,*, Lei Zhou^2,3^, Yun Lai^1,3,6^,*

†These authors contributed equally to this work.

*Corresponding authors

**Affiliations**

^1^School of Physical Science and Technology & Collaborative Innovation Center of Suzhou Nano Science and Technology, Soochow University, Suzhou 215006, China.

^2^State Key Laboratory of Surface Physics and Key Laboratory of Micro and Nano Photonic Structures (Ministry of Education), Fudan University, Shanghai 200433, China.

^3^Collaborative Innovation Center of Advanced Microstructures, Nanjing 210093, China.

^4^Shanghai Engineering Research Center of Ultra-Precision Optical Manufacturing, Green Photonics and Department of Optical Science and Engineering, Fudan University, Shanghai 200433, China.

^5^State Key Laboratory of Applied Optics, Changchun Institute of Optics, Fine Mechanics and Physics, Chinese Academy of Sciences, Changchun 130033, China.

^6^National Laboratory of Solid State Microstructures, School of Physics, Nanjing University, Nanjing 210093, China

**Official email addresses of all authors**

Hongchen Chu, hcchu92@stu.suda.edu.cn; Qi Li, 15110190004@fudan.edu.cn;

Bingbing Liu, bbliu0@stu.suda.edu.cn; Jie Luo, luojie@suda.edu.cn;

Shulin Sun, sls@fudan.edu.cn; Zhi Hong Hang, zhhang@suda.edu.cn;

Lei Zhou, phzhou@fudan.edu.cn; Yun Lai, laiyun@nju.edu.cn

**Contact details of corresponding authors**

Yun Lai, Telephone number: 0086 051265221640.

Shulin Sun, Telephone number: 0086 02165642091.

Zhi Hong Hang, Telephone number: 0086 051265226765.

**This file includes:**

Supplementary text

1. Experimental setup for measuring the electric field distribution of the practical hybrid rhombic cloak

2. Experimental setup for measuring the power radiation pattern

3. Metasurface: the effective medium model

4. Detailed parameters of the metasurfaces and ZIM layers in the cloaks in Figs. 2a-c

5. Analysis on the functionalities of the metasurfaces

6. Power flow distribution in the cloaking shell

7. The design method for wide-angle or omnidirectional cloaking

8. Detailed geometries of the six ABA units and fabrication of the metasurface

9. Double-zero property of the designed photonic crystal

10. Effective medium model for polarization-independent metasurface

11. Divergent parameters in traditional transformation-optics cloaks when $d/R\to0$

12. Numerical simulation methods

13. Caption for Supplementary Movie S1

Supplementary figures S1-9

Figure S1. Schematic picture of the experimental setup.

Figure S2. Transmission phase spectra of an effective medium layer of the highly transparent metasurface.

Figure S3. Derived phase shift and incident angle of each point on metasurfaces in Fig. 2.

Figure S4. Detailed parameters of metasurfaces in Fig. 2.

Figure S5. Functionalities of the metasurfaces in cloaking shells.

Figure S6. Time-averaged power flow in a circular cloak.

Figure S7. Cloaking shells with incidence-dependent parameters.

Figure S8. Detailed geometries of the six ABA units of the metasurface.

Figure S9. Designed double-zero-index medium consisting of dielectric photonic crystals.

Figure S10. Polarization-independent metasurfaces and cloaks.

Other Supplementary Information for this manuscript includes the following:

**Supplementary Movie S1.** Time evolution of electric field distribution for the designed circular cloak in Fig. 2a under illumination of a transverse electric polarized plane wave from the left side.

**1. Experimental setup for measuring the electric field distribution of the practical hybrid rhombic cloak**

We experimentally demonstrate the cloaking effect of the designed practical cloaking shell at microwave frequency with the measurement setup shown in Fig. S1. Two plexiglas plates coated with aluminum foil separated by absorption layers with thickness *d*=12 mm is used to form a parallel plate metallic waveguide. At microwave frequency, a plexiglas plate coated with aluminum foil serves perfectly as a metallic plate. The rhombic cloaking shell and the obstacle are sandwiched by the upper and lower plate of the waveguide. The length, width and thickness of the upper and lower plates are 800×600×2 mm^3^ and800×600×5 mm^3^, respectively. There is a scanning rectangular window of 280×100 mm^2^ on the upper plate so that a dipole antenna can be used to probe the electric field inside the waveguide. The probing antenna and the plate are mounted to a computer controlled translational stage (not shown here). The emitting horn antenna is 2 meters away from the front of the waveguide at the same altitude to guarantee a flat wavefront of the incident wave at the entrance of the waveguide. Both the probing antenna and the horn antenna are connected to a network analyzer (Keysight E5071C) for data acquisition.

2. Experimental setup for measuring the power radiation pattern

In the far-field experiments measuring the power radiation pattern of the metasurface, an emitting horn antenna is placed 1m away from the samples to generate the incident waves. A receiving antenna placed at the same distance is used to measure the radiation pattern. The receiving antenna could be freely moved around the sample so that we could receive scattering signals in all directions. Both the emitting horn antenna and the receiving antenna are connected to a vector network analyzer (Agilent E8362C PNA) for data acquisition. The received signals are normalized against a reference measured when the meta-surface is replaced by a metal plate of the same size.

**3. Metasurface: the effective medium model**

In order to design the desired transparent metasurface with the full range of phase-tuning ability, an effective medium model is applied. Under the TE polarization with electric fields polarized in the $z$ direction (i.e., out-of-plane), the adopted effective medium for the metasurface is an anisotropic one with two independent parameters $\varepsilon_{\text{z}}$ and $\mu_{\text{//}}$, while $\mu_{\perp}$ is set to be unity. Here $\varepsilon_{\text{z}}$ denotes the relative permittivity long z direction. $\mu_{\text{//}}$ and $\mu_{\perp}$ denote the components of the relative permeability tensor along and perpendicular to the surface, respectively. The complex transmission coefficient through such a layer can be obtained as

, (S1)

Where $\alpha$ is the incident angle, $a=\sqrt{\varepsilon_{z}-\sin^{2}(\alpha)/\mu_{\perp}}$, $b=\sqrt{\mu_{\text{//}}}$, , $k_{0}$ is the wave number in space and $d$ is the thickness of the layer. By substituting the condition of $|t|=1$ into Eq. S1, we obtain one of the solutions as

$\mu_{\text{//}}\text{=}(\varepsilon_{z}-\sin^{2}\alpha/\mu_{\perp})/\cos^{2}\alpha$. (S2)

Equation (S2) shows the condition of maintaining a total transmittance under the incident angle $\alpha$. Fig. S2a shows the simulated electric field distribution of an effective medium layer with $\varepsilon_{\text{z}}$, $\mu_{\text{//}}$, $\mu_{\perp}$ and $d$ set as 13, 17, 1, and $0.01\lambda$, respectively, where $\lambda$ is the wavelength in the background medium. The simulation is conducted by using finite element numerical software COMSOL Multi-physics. Under an incident angle of $\alpha=30^{\circ}$, such a layer is capable of producing a phase shift of while $\left|\text{ }t\text{ }\right|\text{=1}$ is maintained. The inset graph shows the zoomed-in view of the layer with fast phase change inside. In Fig. S2b, we set $\mu_{\perp}$ and $d$ as 1 and $0.01\lambda$, and plot the phase discontinuity as a function of the incident angle $\alpha$ and $\varepsilon_{\text{z}}$, which is obtained as $Arg[t]$, under the condition of $\left|\text{ }t\text{ }\right|\text{=1}$ via Eq. S2. Obviously, the phase shift produced by such a transparent metasurface covers the range of $[0,2\pi)$ for all incident angles. The dashed curve in Fig. S2b denotes a contour line of , and the parameter space under the dashed curve is sufficient to cover the range of $[0,2\pi)$for all incident angles. Fig. S2 guides us to design the effective medium model for the metasurface discussed in the main text.

**4. Detailed parameters of the metasurfaces and ZIM layers in the cloaks in Figs. 2a-c**

The metasurfaces in Fig. 2a-c are designed according to the following design principle. Firstly, we derived the required phase shift and the incident angle at each point on the metasurface as shown in Fig. S3. Then we utilize the phase shift diagram shown in Fig. S2b to search for the proper parameters of each point on the metasurfaces. Figures S4a-c show respectively the $\varepsilon_{\text{z}}$ and $\mu_{\text{//}}$ distributions of the metasurface in the circular cloak, the rhombic cloak, and irregular polygon cloak shown in Fig. 2. Here, $\mu_{\perp}$ for all the metasurfaces is fixed at 1. We note that since the cloaks in the first two cases are of bilateral symmetry, we apply the parameters of the metasurfaces on the left side to that on the right side so as to simplify the design procedure and the complexity in simulations.

The thickness of the ZIM layer in Figs. 2a-c are respectively $0.4\lambda$, $0.3\lambda$, $0.2\lambda$.

**5. Analysis on the functionalities of the metasurfaces**

In this section, we take the circular cloaks as an example to illustrate the functionality of the metasurfaces covering on the ZIM. Though normal incident waves can couple into and tunnel through the ZIM, oblique incident waves impinging on the ZIM are completely reflected due to total reflection as shown in Fig. S5a where the incident plane wave impinging on the $\varepsilon\text{=}\mu\text{=}0.001$ ZIM with oblique incident angle is almost completely scattered. By covering a metasurface to the enter side where the incident wave illuminates on the ZIM (e.g. the left side in this circular cloak case), the incident wave is bent to direction normal to the surface of the ZIM and hence enters into the ZIM without being scattered back as shown in Fig. S5b where only the left side of the ZIM is covered by the well-designed metasurface. When the waves enter into the ZIM, the tunneling effect is activated which can maintain almost full transmission. At the exit side (e.g. the right side in this case), the shape of the ZIM can tailor the wavefront of radiation waves, usually destroying the prospect of invisibility as shown in Fig. S5b. Again, we adopt metasurface to recover the wavefront of the emission waves and match the phase delay in the background. In Fig. S5c, the simulated electric field distribution for a circular cloak covering the whole precisely designed metasurface shows perfect wave bending and phase compensation effects and the wave tunneling and emission effects of ZIMs. Moreover, the wave tunneling effect of ZIMs is irrespective of the embedded PMC cavity under a TE polarized illumination, rendering the cloaking effect as shown in Fig. S5d.

**6. Power flow distribution in the cloaking shell**

By making further analysis of the power flow distribution in the ultrathin circular cloaking shells, we find that most of the electromagnetic wave energy incident on the cloak surface is actually conducted through the ZIM via tunneling effect. In Fig. S6a, we show the time-averaged power flow map in space. The flux is concentrated in the very thin layer of the cloak. In the inset zoomed-in view graphs, it is clearly shown that the energy flux is mainly concentrated in the ZIM layer instead of the metasurface. In Fig. S6b, we plot the magnitude of the power flow in the ZIM layer as a function of the angle $\theta$. The flux increases gradually from $\theta=\text{-}\pi$and reaches maximum at $\theta=\pm\pi/2$ and then decreases gradually to zero at $\theta=0$, due to the radiation of wave energy.

**7. The design method for wide-angle or omnidirectional cloaking**

Although the hybrid cloaks proposed above are designed at one particular incident angle, the idea in principle can be extended to omnidirectional cloaking by using coding/programmable or nonlocal metasurfaces, which can exhibit different material parameters for different incident directions. In numerical calculations, we can get the required parameters of every point on the metasurfaces for different incident angles $\alpha$ e.g., $\varepsilon_{z}(\alpha)$ and $\mu_{\text{//}}(\alpha)$. Here, we take the circular cloak (see Fig. 2a) for example again. The required phase discontinuity of the metasurface is , where $R$ is the radius, and $c_{1}\text{=}2m\pi\text{+}k_{0}R$, where $m$ is an arbitrary integer. $\theta$ denotes the tangential position of the metasurface as shown in Fig. S7a, which is equal to incident angle $\alpha$ in the analysis. Thus, we have $\theta=\alpha\text{=}\arcsin\left(k_{\text{//}}/k_{0}\right)$ with $k_{\text{//}}$ being the component of wave vector parallel to the metasurface. In addition, the phase change through the metasurface can be written as with $t$ as described in Eq. S1. By assuming , we have

. (S3)

Combining Eqs. S2 and S3, we obtain $\varepsilon_{z}(\alpha)$ and $\mu_{\text{//}}(\alpha)$ for every point on the metasurface as a function of $\alpha$. With known the required $\alpha\text{-}$ dependent parameters of the metasurfaces, the remaining work is to design coding/programmable or nonlocal metasurfaces with such unique parameters, which is a quite difficult but not impossible work, as proved by some previous works (30-33). For a circular cloak with $R=2\lambda$ as shown in Fig. S7a (under incidence from the left), the calculated $\alpha\text{-}$ dependent parameters of the metasurfaces are plotted in Fig. S7b. The required effective parameters of this nonlocal metasurface for $\alpha\text{=-45}^{\circ}$ and $\alpha\text{=-90}^{\circ}$ incident waves can be obtained from Fig. S7b. The perfect cloaking effects for incidences from the left bottom and from the bottom are demonstrated by numerical simulations, as shown in Figs. S7c and S7d, respectively.

**8. Detailed geometries of the six ABA units and fabrication of the metasurface**

We designed six unit cells by adopting ABA sandwich structures as the building block of the high efficiency metasurface, where A and B layers are carefully designed 0.018 mm-thick metallic micro-structures separated by 1.5mm-thick dielectric spacer ($\varepsilon\text{=}4.3$). Figs. S8a-f show the detailed geometrical parameters of A and B layers in the six designed unit cells. Fig. S8g shows the photo of a part of the fabricated metasurface with the red frame marking the six unit cells as one super cell.

The metasurface are fabricated by laminating two 1.5-mm-thick FR4 printed circuit boards. The two boards are separated by a 0.018 mm-thick copper patterns of Layer B and are covered by two Layer A, with the same thickness but distinct pattern according to theoretical design. The relative permittivity of the dielectric boards is $\varepsilon_{r}=4.3$.

**9. Double-zero property of the designed photonic crystal**

In this section, we introduce the detailed design of the photonic crystal (PC) discussed in the main text. It is a 2D square lattice structure constructed of cylinders with radius $r=0.226a$, where *a*=17.46 mm is lattice constant, relative permittivity $\varepsilon=8.1$ and relative permeability $\mu=1$. The numerically calculated band structure of this PC exhibits a Dirac cone at the center of the Brillouin zone ($\Gamma$ point, where $\text{k}=0$) at 10.2 GHz as shown in Fig. S9a and S9b. Full wave simulations are carried out to demonstrate the double-zero property of this photonic crystal. As shown in the upper panel Fig. S9c, the incident wave from the left side tunnels through 6 layers of photonic crystals. For comparison, effective medium with $\varepsilon=\mu=0.001$ of the same thickness is simulated and the simulated results shown in the lower panel agree well with that in the upper panel, demonstrating the near zero property of the designed photonic crystals. We also calculated the transmittance of this photonic crystals with different number of layers. As show in Fig. S9d, the transmittance is found to be unity and the transmittance of photonic crystals agrees well with that of the effective medium with $\varepsilon=\mu=0.001$ and the same thickness. We then consider a 14×14 array of the designed PC filling in the corner of a $90^{\circ}$ bending waveguide as shown in Fig. S9e under illumination of a TE plane wave from the left. From the simulation results shown in Fig. S9e we observe that the incident wave tunnels through the bending waveguide via PC with unnoticeable phase accumulation inside PC, which means an infinite wavelength, which is the unique character of ZIMs. For comparison, we then replace the designed 2D PC by a homogeneous ZIM with $\varepsilon\text{=}\mu\text{=}0.001$. The same tunneling effect and unnoticeable phase accumulation inside the ZIM are also observed in the simulation results shown in Fig. S9f. The good agreement between Fig. S9e and Fig. S9f indicates that the designed PC can function as ZIM at the designed frequency (10.2 GHz).

**10. Effective medium model for polarization-independent metasurface**

Though up to now we mainly discussed about cloaks for TE or TM polarization separately, as long as the parameters of metasurface and ZIMs are designed properly, they are able to work under both TE and TM polarizations. To design cloaks valid for both TE and TM polarized incidence, the ZIM are required to be double zero index guaranteeing that both TE and TM polarized waves can tunnel through it. In addition, metasurfaces in those cloaks are also need to be sufficient for both two polarizations. By utilizing effective medium model, we design a circular cloak with the same size as that in Fig. 5a that works for both TE and TM polarized incidence. Metasurfaces with $\varepsilon_{z}$, $\mu_{\text{//}}$ shown in Fig. S4a and $\mu_{\perp}\text{=1}$ and $\mu_{z}\text{=}\varepsilon_{z}$, $\varepsilon_{\text{//}}\text{=}\mu_{\text{//}}$, $\varepsilon_{\perp}\text{=}\mu_{\perp}$ are sufficient to generate the phase distribution shown in Fig. S3a for both TE and TM polarizations. Numerical simulations are carried out to study cloaks composed of this metasurface and double-zero ZIM with $\varepsilon\text{=}\mu\text{=0.001}$. Good cloaking effects are observed in simulated results shown in Fig. S10b and Fig. S10c demonstrating that this cloak works quite well for both TE and TM polarizations.

11. Divergent parameters in traditional transformation-optics cloaks when$d/R\to0$

When $d/R\to0$, where $d$ is the thickness of the cloak and $R$ is the radius of the cloaked area, transformation-optics cloaks require divergent parameters in the cloaking shell. For example, the relative permittivity and permeability of a circular cloaking shell (e.g. under TM polarization) are $\varepsilon_{\perp}=\frac{r-R}{r}$, $\varepsilon_{\text{//}}=\frac{r}{r\text{-}R}$, $\mu_{z}=(\frac{R}{d}+1)^{2}\frac{r-R}{r}$, where $r$ is the radial coordinate ($R<r<R+d$). $\varepsilon_{\perp}$, $\varepsilon_{\text{//}}$ denote the permittivity perpendicular and parallel to the shell surface in the *xy* plane, and $\mu_{z}$ denotes the permeability in the *z* direction, respectively. Clearly, when $d/R\to0$, $\mu_{z}$ and $\varepsilon_{\text{//}}$ both approaches infinity. Even for the reduced parameters, i.e. $\varepsilon_{\perp}=\text{（}\frac{r-R}{r}\text{）}^{2}$, $\varepsilon_{\text{//}}=1$, $\mu_{z}=(\frac{R}{d}+1)^{2}$, $\mu_{z}$ still approaches infinity when $d/R\to0$. These requirements make it difficult to realize ultrathin cloaks by transformation optics, especially in the optical frequency regime.

12. Numerical simulation methods

Cloaking shells with metasurface modeled by the effective medium layer are simulated by a commercial finite element solver package – COMSOL Multiphysics. Some complicate shapes in the simulations are firstly built in AutoCAD and then imported into COMSOL. The incident plane wave is excited by a background electric field in the form of $E_{b}=\exp(i(k_{0}\cos(\alpha)x+k_{0}\sin(\alpha)y))$. Perfectly matched layers are set around the region to absorb the scattering waves. The spatially varying parameters of the metasurface are set by utilizing the interpolation function in COMSOL. The practical transparent metasurface and rhombic cloaking device are simulated in a 3D electromagnetic simulation software – CST. Open boundary condition is set around the simulation region to absorb the scattering waves.

**13. Caption for Supplementary Movie S1**

Time evolution of electric field distribution for the designed circular cloaks in Fig. 2a under illumination of a transverse electric polarized plane wave from the left side. The outer radius of the cloak is $2\lambda$. The thickness of the metasurface is $0.01\lambda$. The $\varepsilon_{\text{z}}$ and $\mu_{\text{//}}$ distributions of the metasurface are shown in Fig. S4a while $\mu_{\perp}$ is set as 1. The thickness of the ZIM layer with $\varepsilon\text{=}\mu\text{=}0.001$ is $0.4\lambda$.

Supplementary Figures


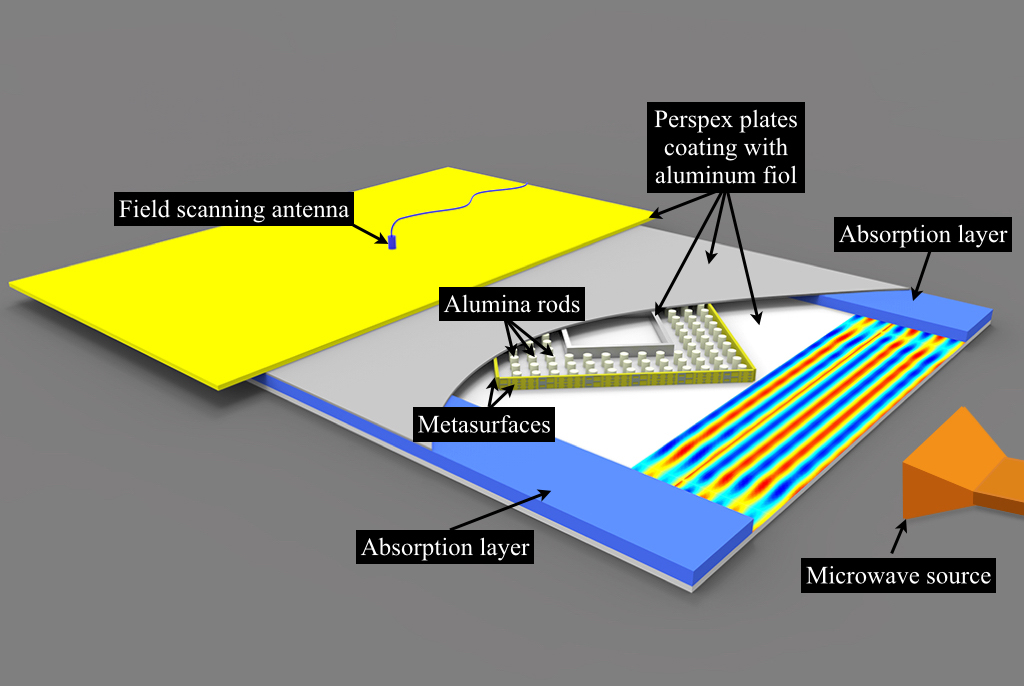


**Figure S1.** Schematic picture of the experimental setup.


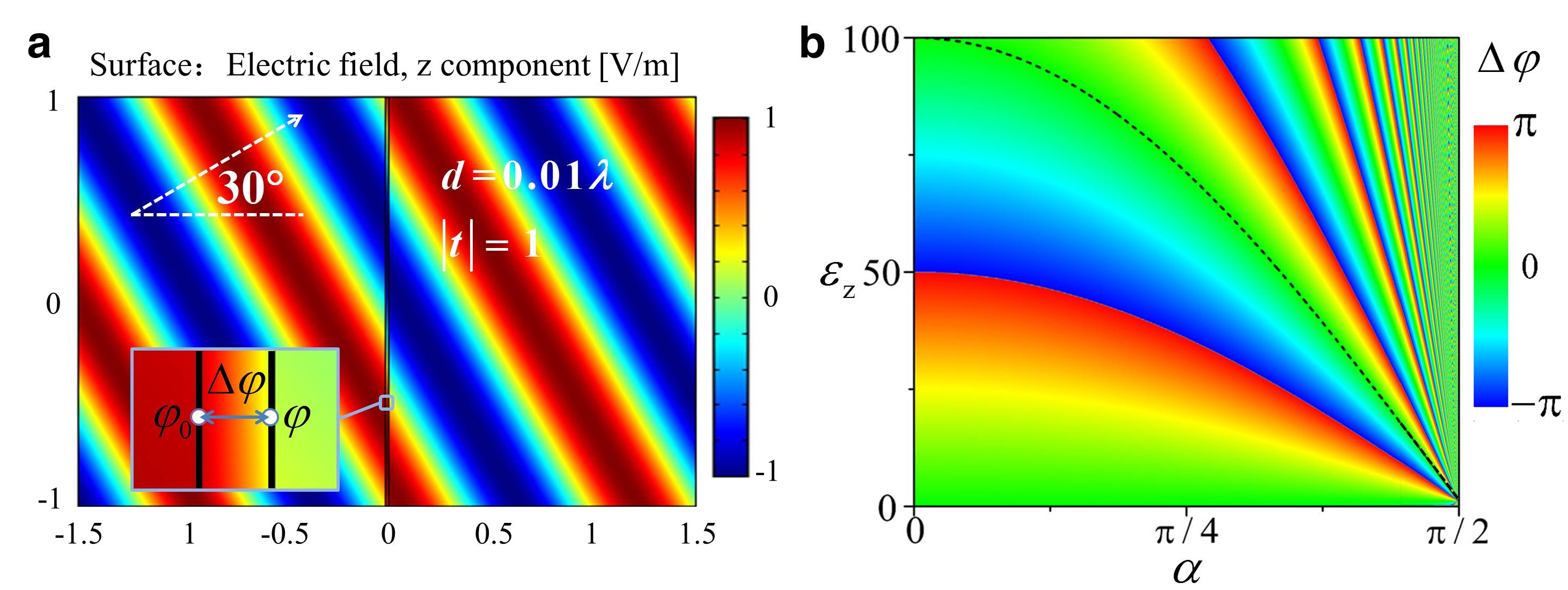


**Figure S2.** Transmission phase spectra of an effective medium layer of the highly transparent metasurface. (**a**) Simulated electric field distribution for the effective medium layer with a thickness of $d=0.01\lambda$ under a TE-polarized plane wave with incident angle $\alpha=30^{\circ}$, where $\lambda$ is the wavelength in vacuum. $\varepsilon_{\text{z}}$, $\mu_{\text{//}}$ and $\mu_{\perp}$ of the layer are 13, 17 and 1, respectively. The transmission amplitude $t$ is found to be unity due to the impedance matching. (**b**) The phase shift discontinuity as a function of incident angle $\alpha$ and $\varepsilon_{z}$while $d$ is fixed at $0.01\lambda$. The dashed curve indicates the minimum $\varepsilon_{\text{z}}$ required to cover the range of $[0,2\pi)$. The premise of $|t|=1$ is guaranteed by Eq. S2.


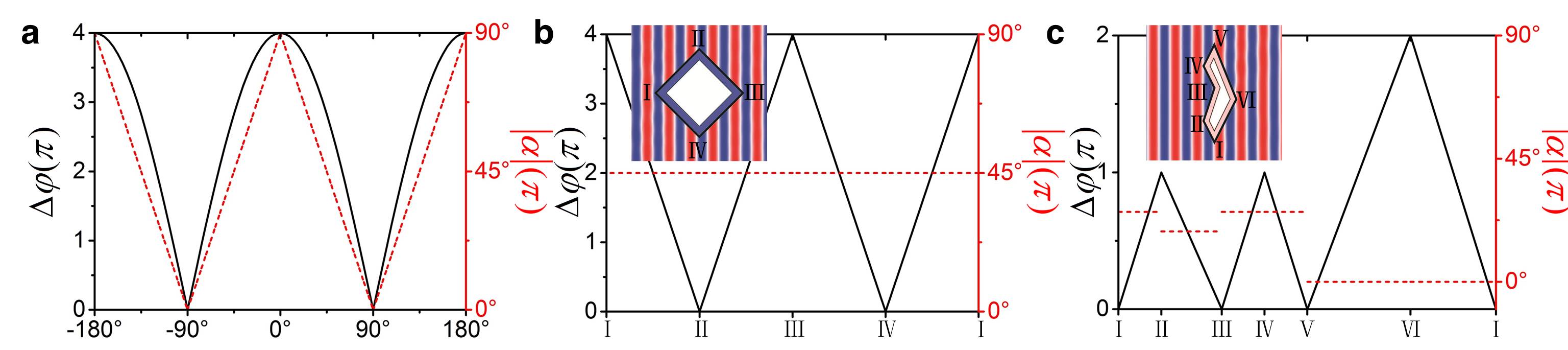


**Figure S3****.** Derived phase shift and incident angle of each point on metasurfaces in Fig. 2. Derived phase shift and incident angle of each point on metasurfaces in the simulation models of the circular cloak (Fig. 2**a**), rhombic cloak (Fig. 2**b**) and irregular polygon cloak (Fig. 2**c**) in the main text, respectively. Black solid lines and red dashed lines represent phase shifts and incident angles, respectively. The Roman numerals from Ⅰ to Ⅳ (from Ⅰ to Ⅵ) represent the four (six) corners of the metasurface as shown in the inset in Fig. S3b (Fig. S3c).


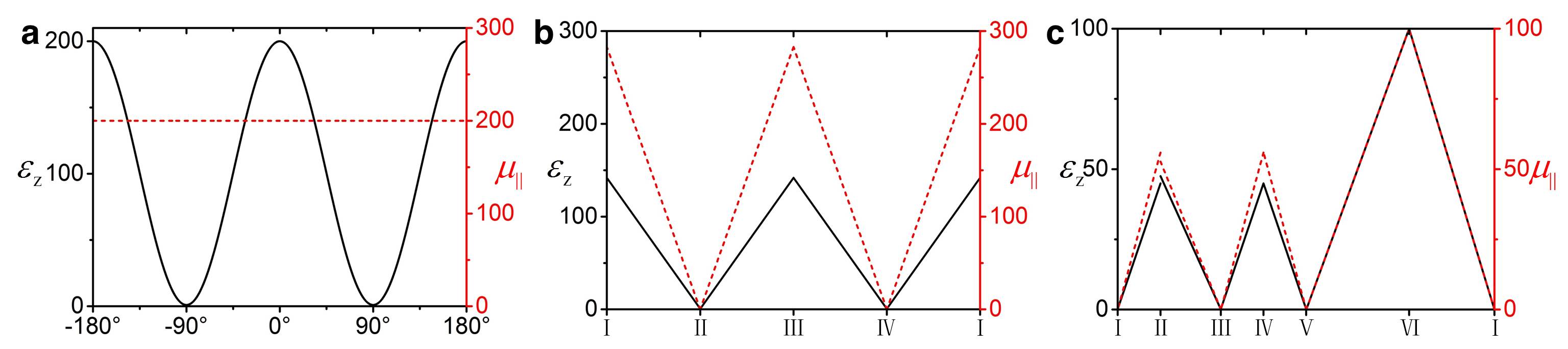


**Figure S4.** Detailed parameters of metasurfaces in Fig. 2. $\varepsilon_{\text{z}}$ and $\mu_{\text{//}}$ distributions of metasurfaces shown in (**a**), (**b**) and (**c**) relate to the simulation models of the circular cloak (Fig. 2a), rhombic cloak (Fig. 2b) and irregular polygon cloak (Fig. 2c) in the main text, respectively. Black solid lines and red dashed lines represent $\varepsilon_{\text{z}}$ and $\mu_{\text{//}}$, respectively.


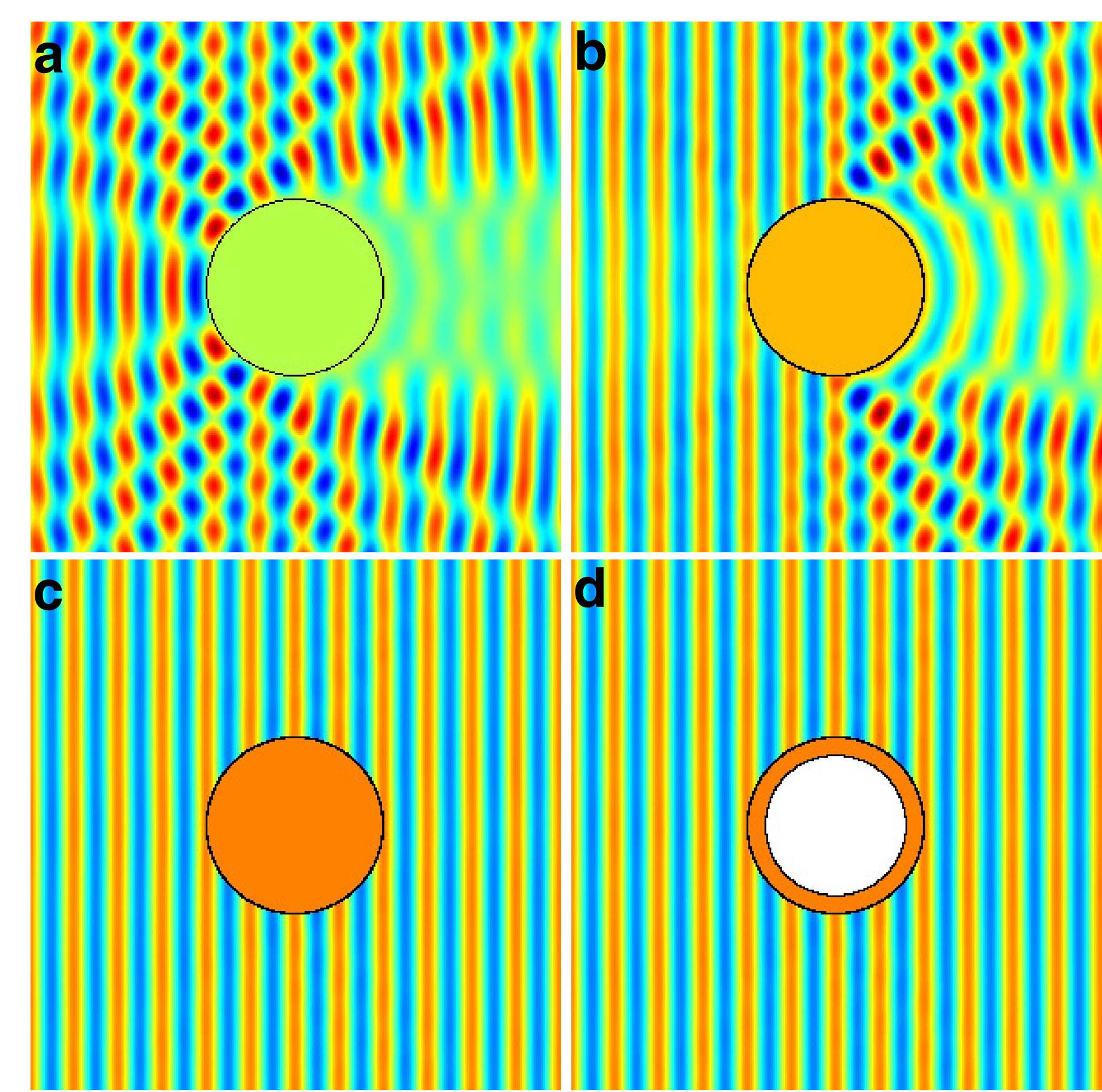


**Figure S5.** Functionalities of the metasurfaces in cloaking shells. Snapshots of the total electric fields for (**a**) A circular ZIM with $\varepsilon\text{=}\mu\text{=}0.001$ under the illumination of a TE plane wave from the left scatters almost all the incident wave. (**b**) Only the left side of this circular ZIM is covered with the precisely designed metasurface. The incident wave enters ZIM. On the right side the direction of the emitting wave is normal to the surface of ZIM. (**c**) Both left and right sides of ZIM are covered with the well-designed metasurface. The incident wave passed the metasurface and ZIM without any scattering. (**d**) PMC obstacles inside ZIM do not affect the wave bending and tunneling effect, rendering the cloaking effect.


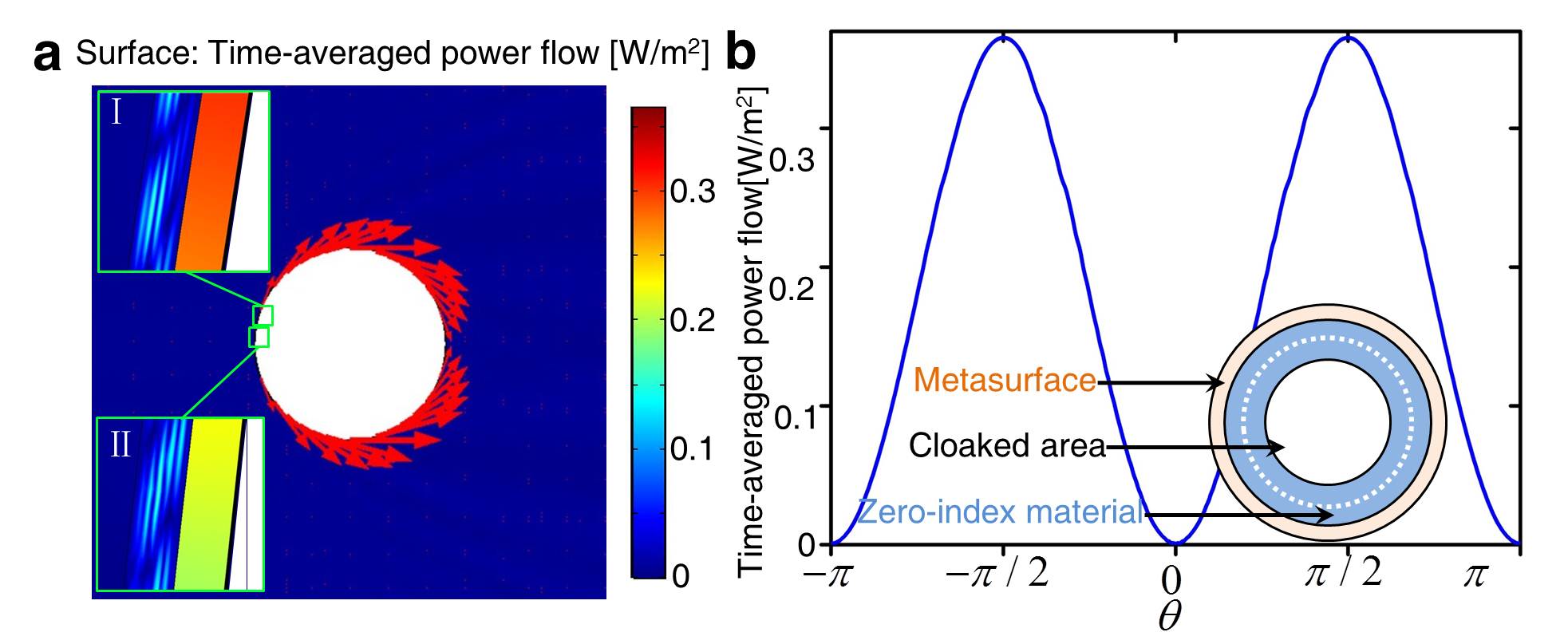


**Figure S6.** Time-averaged power flow in a circular cloak. (**a**) Time-averaged power flow distribution for the circular cloak show in Fig. 5b. The orientation of red arrows represents the direction of power flow and the arrow length is proportional to the magnitude. Insets show the zoomed-in view of the narrow ZIM and the coating metasurface. (**b**) The magnitude of the time-averaged power flow in the very thin ZIM layer (the dashed curve shown in the inset graph) as a function of angle $\theta$.


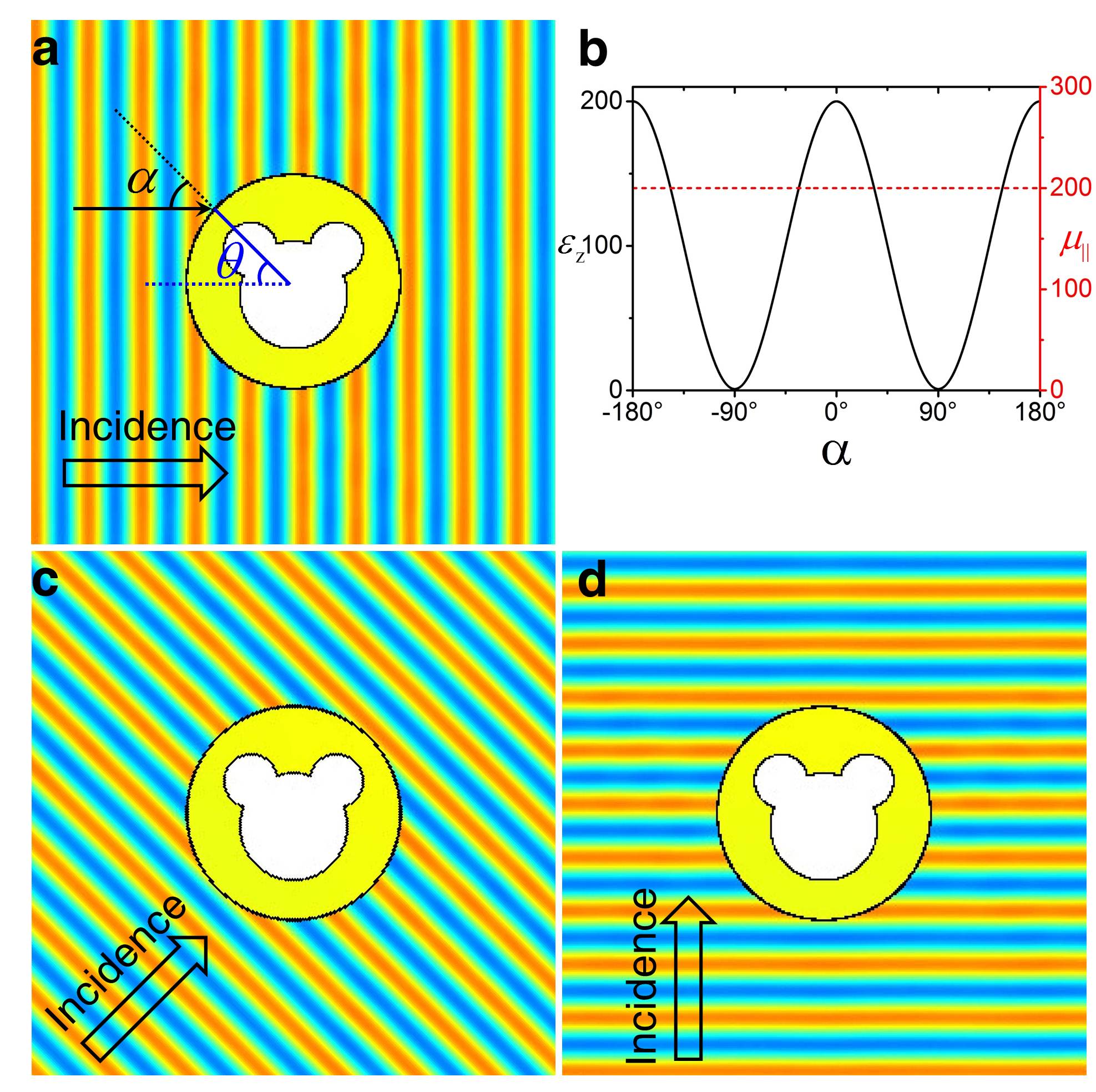


**Figure S7.** Cloaking shells with incidence-dependent parameters. (**a**) Simulated electric distribution for circular cloaks composed of ZIM and nonlocal metasurfaces illuminated by TE polarized plane wave at incident angle $\theta=0^{\circ}$ (**b**). Calculated $\alpha\text{-}$ dependent parameters of the metasurfaces for circular cloaks in (a). (**c, d**) Incident waves impinge on the cloaks with $\theta=\text{-45}^{\circ}$ and $\theta=\text{-90}^{\circ}$. Perfect omnidirectional cloaking effect is observed.


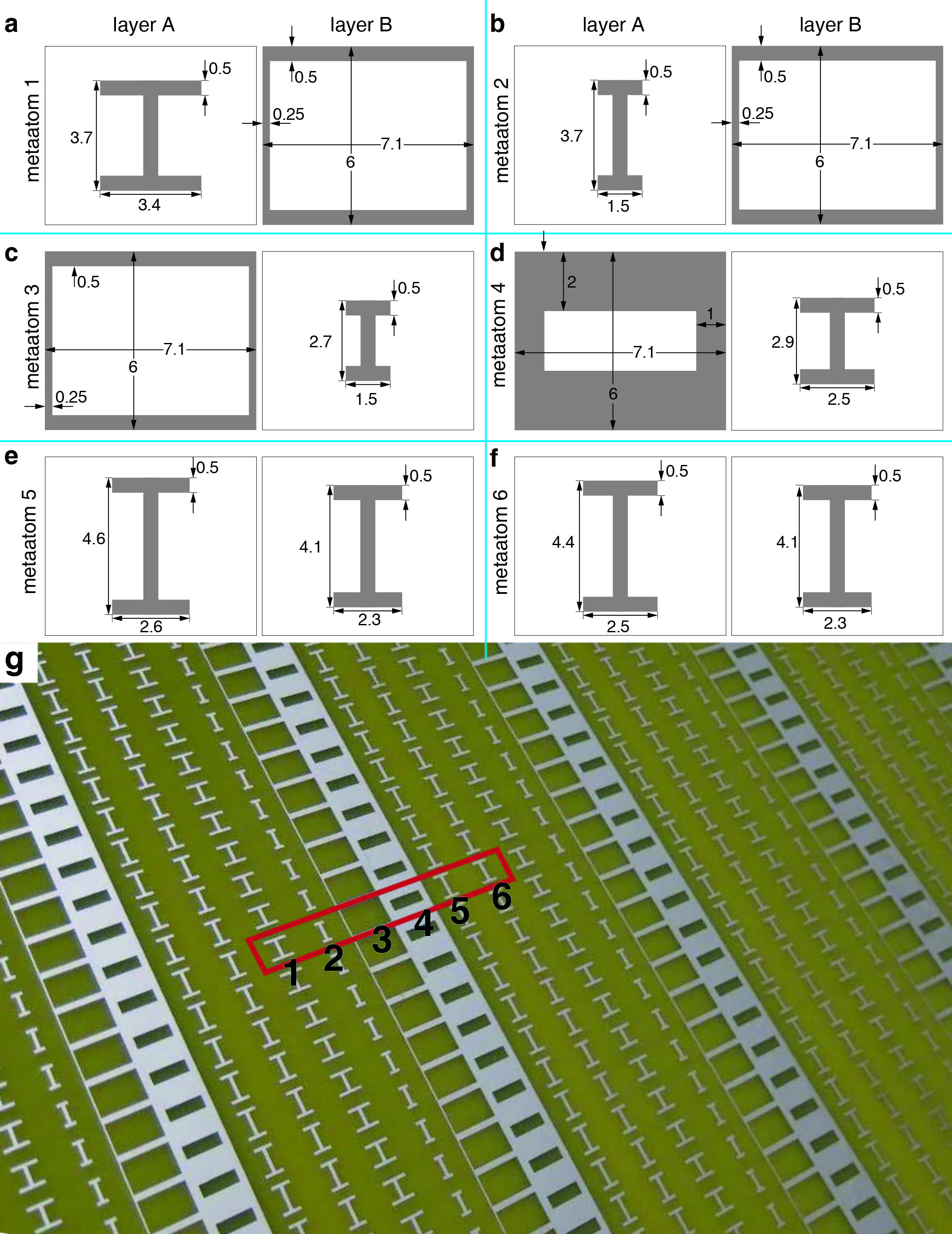


**Figure S8.** Detailed geometries of the six ABA units of the metasurface. (**a-f**) Geometrical parameters of coppery patterns in A and B layers of six designed metasurface units. The gray color depicts copper which is perfect electric conductor in the microwave regime. Unit of these numbers listed in the insets is mm. (**g**) Photo of part of the fabricated metasurface. The red rectangle marks one super cell of the metasurface.


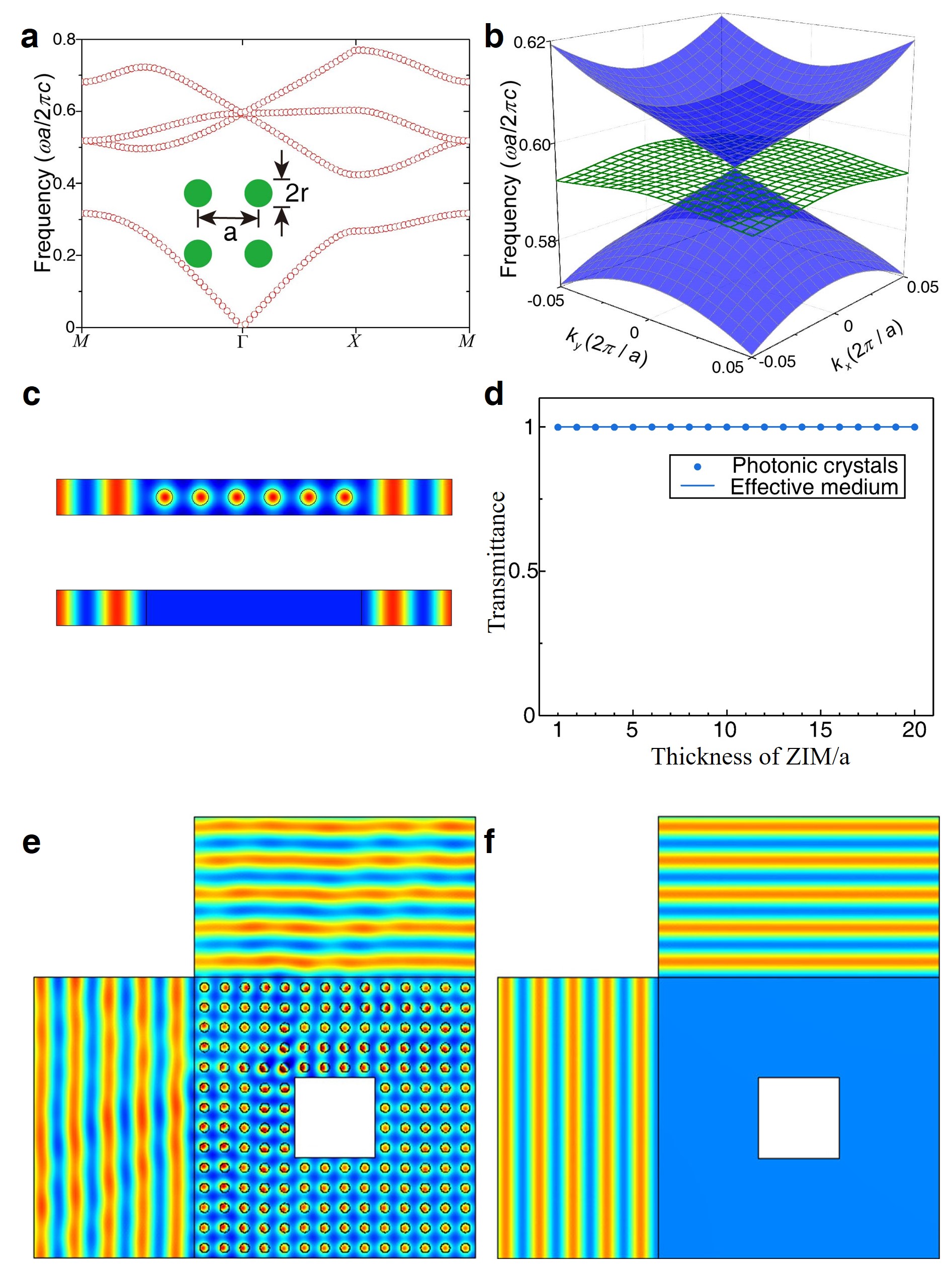


**Figure S9.** Designed double-zero-index medium consisting of dielectric photonic crystals. (**a**) Band diagrams for a 2D square lattice PC constructed of cylinders with relative permittivity $\varepsilon=8.1$, relative permeability $\mu=1$ and radius $r=0.226a$, where *a*=17.46 mm is the lattice constant. (**b**) Dispersion surfaces near the Dirac point frequency of the band structure shown in (a). (**c**) Simulated electric field distribution for 6 layers photonic crystals (upper panel) and effective medium with $\varepsilon\text{=}\mu\text{=0.001}$ and thickness of $d=6a$ (lower panel). (**d**) Transmittance of photonic crystals with different numbers of layers (blue circles). The blue solid line depicts the transmittance of $\varepsilon\text{=}\mu\text{=0.001}$ effective medium with different thickness. (**e**) Simulated electric field distribution for a $90^{\circ}$ bending waveguide whose corner is filled with 14×14 PCs with a 4*a*×4*a* PMC barrier inside under illumination of TE polarized waves from the left side. The incident wave tunnels through the bending waveguide and propagates upward with relatively flat wave fronts and negligible disturbance. (**f**) Simulated electric field distribution for a $90^{\circ}$ bending waveguide whose corner is filled with $\varepsilon\text{=}\mu\text{=0.001}$ ZIM with a 4*a*×4*a* PMC barrier inside under illumination of TE polarized waves from the left side.


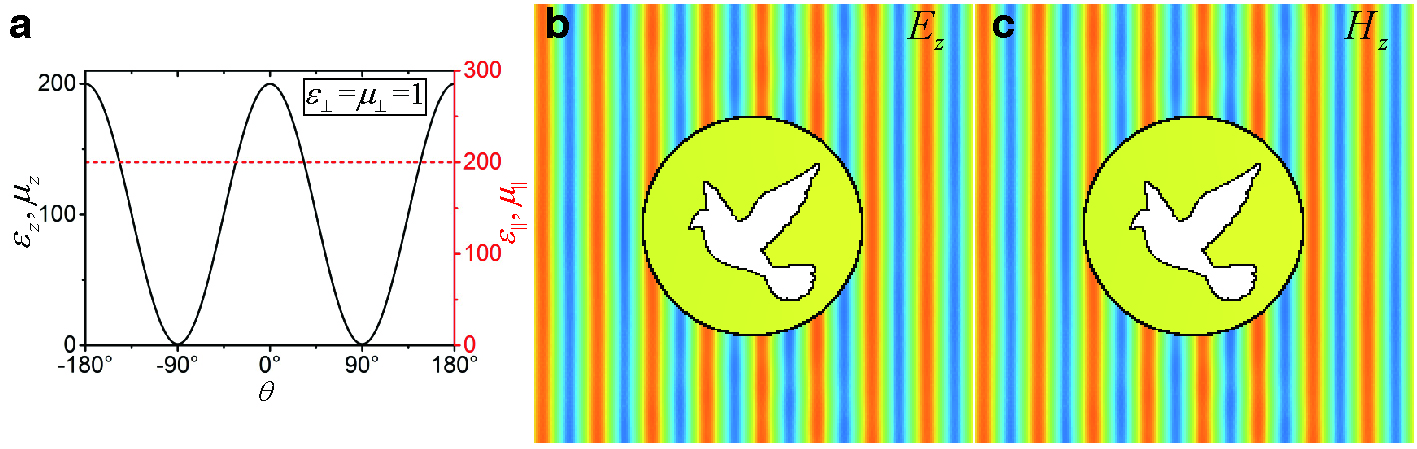


**Figure S10.** Polarization-independent metasurfaces and cloaks. (**a**) Effective parameters distribution of a metasurface which can generate phase shift distribution shown in Fig. S3a under both TE and TM polarizations. (**b**) Electric field distribution for a cloak composed of metasurface with parameters shown in (a) and the ZIM with $\varepsilon\text{=}\mu\text{=0.001}$ under illumination of a TE polarized plane wave. Here a dove shaped PMC and space inside are cloaked. (**c**) Magnetic field distribution for the same cloak under illumination of a TM polarized plane wave. Here a dove shaped PEC and space inside are cloaked.
